# Whole transcriptome analysis to explore the impaired immunological features in critically ill elderly patients with sepsis

**DOI:** 10.1186/s12967-023-04002-z

**Published:** 2023-02-23

**Authors:** I-Chieh Chen, Hsin-Hua Chen, Yu-Han Jiang, Tzu-Hung Hsiao, Tai-Ming Ko, Wen-Cheng Chao

**Affiliations:** 1 Department of Medical Research, Taichung Veterans General Hospital, Taichung, Taiwan; 2 Division of General Internal Medicine, Department of Internal Medicine, Taichung Veterans General Hospital, Taichung, Taiwan; 3 Big Data Center, National Chung Hsing University, Taichung, Taiwan; 4 Department of Industrial Engineering and Enterprise Information, Tunghai University, Taichung, Taiwan; 5 Institute of Biomedical Science and Rong Hsing Research Centre for Translational Medicine, National Chung Hsing University, Taichung, Taiwan; 6 Department of Public Health, Fu Jen Catholic University, New Taipei City, Taiwan; 7 Institute of Genomics and Bioinformatics, National Chung Hsing University, Taichung, Taiwan; 8 Department of Biological Science and Technology, National Yang Ming Chiao Tung University, Hsinchu, Taiwan; 9 Institute of Bioinformatics and Systems Biology, National Yang Ming Chiao Tung University, Hsinchu, Taiwan; 10 Institute of Biomedical Sciences, Academia Sinica, Taipei, Taiwan; 11 Department of Critical Care Medicine, Taichung Veterans General Hospital, No. 1650 Taiwan Boulevard, Section 4, Xitun District, Taichung City, 40705 Taiwan; 12 Department of Post-Baccalaureate Medicine, College of Medicine, National Chung Hsing University, Taichung, Taiwan; 13 Department of Automatic Control Engineering, Feng Chia University, Taichung, Taiwan

**Keywords:** Sepsis, RNA-Seq, Elderly ICU patient, Differentially expressed genes, T-cell receptor

## Abstract

**Background:**

Sepsis is a frequent complication in critically ill patients, is highly heterogeneous and is associated with high morbidity and mortality rates, especially in the elderly population. Utilizing RNA sequencing (RNA-Seq) to analyze biological pathways is widely used in clinical and molecular genetic studies, but studies in elderly patients with sepsis are still lacking. Hence, we investigated the mortality-relevant biological features and transcriptomic features in elderly patients who were admitted to the intensive care unit (ICU) for sepsis.

**Methods:**

We enrolled 37 elderly patients with sepsis from the ICU at Taichung Veterans General Hospital. On day-1 and day-8, clinical and laboratory data, as well as blood samples, were collected for RNA-Seq analysis. We identified the dynamic transcriptome and enriched pathways of differentially expressed genes between day-8 and day-1 through DVID enrichment analysis and Gene Set Enrichment Analysis. Then, the diversity of the T cell repertoire was analyzed with MiXCR.

**Results:**

Overall, 37 patients had sepsis, and responders and non-responders were grouped through principal component analysis. Significantly higher SOFA scores at day-7, longer ventilator days, ICU lengths of stay and hospital mortality were found in the non-responder group, than in the responder group. On day-8 in elderly ICU patients with sepsis, genes related to innate immunity and inflammation, such as *ZDHCC19*, *ALOX15, FCER1A*, *HDC, PRSS33,* and *PCSK9*, were upregulated. The differentially expressed genes (DEGs) were enriched in the regulation of transcription, adaptive immune response, immunoglobulin production, negative regulation of transcription, and immune response. Moreover, there was a higher diversity of T-cell receptors on day-8 in the responder group, than on day-1, indicating that they had better regulated recovery from sepsis compared with the non-response patients.

**Conclusion:**

Sepsis mortality and incidence were both high in elderly individuals. We identified mortality-relevant biological features and transcriptomic features with functional pathway and MiXCR analyses based on RNA-Seq data; and found that the responder group had upregulated innate immunity and increased T cell diversity; compared with the non-responder group. RNA-Seq may be able to offer additional complementary information for the accurate and early prediction of treatment outcome.

**Supplementary Information:**

The online version contains supplementary material available at 10.1186/s12967-023-04002-z.

## Introduction

Sepsis is a clinical syndrome that is potentially life-threatening. It is produced by a disordered host response to infection and is a highly heterogeneous and lethal global health threat, particularly in the aged population [1, 2]. Previous studies have found that elderly patients are at a higher risk for sepsis and have higher disease severity than those in the general population [3]. The number of elderly patients who are admitted to the intensive care unit (ICU) due to sepsis has been steadily rising in the past two decades worldwide due to the constant rise in the number of elderly individuals [4]. Notably, despite a high intensity of care, mortality remains high in elderly patients admitted to the ICU, but studies addressing the biological pathways relevant to treatment response in elderly patients with sepsis are still lacking [5].

The immune system of the elderly undergoes numerous biological changes as they age., which influence the elderly immune response, resulting in greater likelihood of immunocompromised conditions [6]. Compromised adaptive immunity is considered a crucial feature of immunosenescence in the elderly [7, 8], but transcriptomic features during sepsis in the elderly remain unexplored. These changes determine not only the susceptibility to bacterial infections but also sepsis progression and clinical outcomes [6]. Therefore, it is critical to investigate the varying immunological responses and mortality-relevant biological features in elderly patients admitted to the ICU [9, 10].

Sepsis is highly heterogeneous and characterized by complex and dynamic immunologic reactions, which involve an initial excessive inflammatory response followed by dysregulated/exhausted adaptive immunity [11]. A number of studies have shown altered adaptive immunity after sepsis; so-called immunoparalysis appears to be one of the key immunological features that leads to persistent compromised immunity in patients with sepsis [12].

RNA-Seq was used in previous studies to find the transcriptomic signature, or “subendotype,” in patients with sepsis. The subendotypes that have been found to be associated with sepsis are early improvement of organ dysfunction after sepsis, dampened adaptive immunity among immunocompromised patients, and responsiveness to steroids in septic shock patients [13, 14]. Utilizing RNA-Seq data, analytical tools, such as MiXCR, have recently been developed to quantify the diversity of T-cell receptors (TCRs) [15, 16]. In this study, we investigated the dynamic transcriptome of septic patients by examining paired samples from day-1 and day-8 and found that immunocompromised septic patients had decreased T cell diversity and compromised T cell function. This study aimed to explore mortality-relevant biological features and transcriptomic features, in order to gain a better understanding of the adaptive immunity-associated signaling in elderly patients who were admitted to the ICU for sepsis.

## Materials and methods

### Study design and data collection

The study was approved by the Institutional Review Board of the TCVGH (IRB no. CE20069B), and informed consent was obtained from all participants. Thirty-seven elderly ICU patients were recruited from Taichung Veterans General Hospital (TCVGH) from Dec 2018 to Jan 2022. At the time of enrollment, demographic information was gathered, and baseline blood samples were taken on day-1 and day-8. In order to preserve the transcriptome, two 4 ml aliquots of blood were collected in sterile EDTA vacutainers and immediately transferred to RNAse-free vials containing 10.5 ml RNAlater^®^ (ThermoFisher, Waltham, MA, USA).

### RNA extraction and sequencing

In this study, we extracted RNA with the PAXgene Blood RNA Kit, and the average RNA integrity number (RIN) was 8.31 ± 0.58. The manufacturer's instructions were followed when building the library, and 1,000 ng of fragmented RNA was used in subsequent experiments. 150-bp paired-end reads were used for RNA-Seq on the NovaSeq platform (Illumina, Inc., San Diego, CA, United States), and each sample had at least 50–60 million reads. The RNA-seq dataset was deposited in the National Center for Biotechnology Information (NCBI) Gene Expression Omnibus (GEO) under accession number GSE216902. The dataset was used for cell proportions and pathway analysis.

### Sequential organ failure assessment (SOFA) score

The sequential organ failure assessment (SOFA) score is a numerical value of sepsis-related organ failure, i.e., it quantifies the number and severity of failed organs. Each score ranges from 0 to 4, with a higher score indicating worsening organ dysfunction [17]. We used the dynamic change in SOFA scores to define responders (R) and non-responders (NR), and a participant was classified as NR if the decrease in SOFA score from day-1 to day-7 was less than 2 [13, 18, 19].

### Bioinformatics analyses of RNA-Seq data

The sequencing was of high quality, Phred scores of 30 were used, and HISAT2 mapped the sequence reads to the reference genome (GRCh38/hg38) [20]. The R package DEseq2 [21] was used to identify the differentially expressed genes (DEGs), and the read counts were calculated using featureCounts [22]. The average mapping rate and read counts were 87.6 ± 4.8% and 65.5 ± 17.6 million reads, respectively. The gprofiler [23] R package was utilized to carry out functional enrichment and pathway analysis on the basis of the Genomes (KEGG, https://www.genome.jp/kegg/) and Gene Ontology (GO, http://geneontology.org/) databases. A corrected *p* value of less than 0.05 was deemed to be significantly enriched. All differentially expressed genes were functionally annotated using Gene Set Enrichment Analysis (GSEA) [24], and a Cytoscape 3.9.0 [25] visualized enrichment map was created. We visualized GSEA results by employing an enrichment map, which organizes gene sets into a similarity network. The node, link, and node color represent the gene-set, the overlap of member genes, and the enrichment score [24], respectively.

### Diversity of TCR analyses

RNA-Seq data were used in MiXCR v3.0.13 [15, 16] to quantify the clonotypes of sepsis patients based upon a previously reported protocol [14]. VDJTools v 1.2.1 [26] software was utilized to calculate the counts of complementarity-determining region-3 (CDR3) and sample diversity following the acquisition of the quantitated clonotypes.

### Statistical analyses

We used the Kolmogorov–Smirnov test to check the normality and found that the distribution of a number of variables, including ventilator day, ICU length of stay and hospital length of stay, were left-skewed. To present the data in the unified format, we presented categorical variables frequencies (percentages) for categorical variables and continuous variables as median (interquartile range, IQR). Differences between two groups were analyzed using the Mann–Whitney U test for continuous variables and Fisher’s exact test for categorical variables. The level of significance was set at 0.05, and statistical analyses were two-sided. R version 4.1.0 was used to conduct all of the data analyses.

## Results

### Patient characteristics and SOFA scores

In this study, we enrolled 37 elderly patients with sepsis [median age 83 years (IQR: 79–87 years)] (Table 1). With principal component analysis, we observed that a divergence between day-1 and day-8 was apparently better in the responsive patients (responder group, R) than in the nonresponsive patients (non-responder group, NR), indicating regulated recovery from sepsis in the immunocompetent group (Additional file 1: Fig. S1). Overall, among the 37 patients, there were 23 responsive patients (responder group, R) with median age 82 years (IQR: 79.5–86.5 years) and 14 nonresponsive patients (non-responder group, NR) with median age 84 years (IQR: 79–88 years). Table 1 shows the basic characteristics of the study group. Higher albumin (median 3.3 mg/dl, IQR: 3–3.7 mg/dl) and lower C-reactive protein (median 9.16 mg/dl, IQR: 3.9–17.5 mg/dl) were observed in the treatment than in their counterparts. Compared with the responder group, the non-responder group had significantly higher SOFA scores at day-7, longer ventilator days, longer ICU lengths of stay, and higher hospital mortality.

**Table 1:** Characteristics of elderly ICU patients with sepsis

Variables	All-Patients (n = 37)	Responders (n = 23)	Non-Responders (n = 14)	*P*-value^a^
Demographic data				
Age (years)	83 (79–87)	82 (79.5–86.5)	84 (79–88)	0.754
Gender (Men)	21 (57%)	13 (57%)	8 (57%)	> 0.999
Severity scores				
APACHE II	27 (25–32)	27 (26–32)	27 (22.8–32)	0.975
SOFA score, day-1	11 (8–13)	11 (9–13.5)	11.5 (8–13)	0.801
SOFA score, day-3	9 (7–12)	8 (6–11.5)	9.5 (8.3–13)	0.127
SOFA score, day-7	7 (5–9)	5 (4–7.5)	9 (7–12)^b^	**0.017**
Comorbidities				
Diabetes mellitus	7 (19%)	4 (17%)	3 (21%)	> 0.999
Congestive heart failure	7 (19%)	5 (22%)	1 (7%)	0.687
COPD	4 (11%)	4 (17%)	0 (0%)	0.276
End-stage renal disease	9 (24%)	6 (26%)	3 (21%)	> 0.999
Cerebral vascular disease	7 (19%)	5 (22%)	2 (14%)	0.687
Laboratory data				
White blood cell count (/ml)	11,760 (8670–14,160)	12,720 (11,120–14,155)	10,680 (7455–13,615)	0.199
High Platelet count (1000/ml)	253 (148–355)	289 (197.5–367.5)	202.5 (120.8–312.8)	0.294
Low Platelet count (1000/ml)	75 (27–122)	80 (33.5–149)	44.5 (23.5–111.3)	0.260
Lactate(mg/dl)	32.7 (21.3–60.2)	32.7 (21.4–61.3)	33.8 (19.5–51.1)	> 0.999
Albumin(mg/dl)	3.3 (3–3.7)	3.3 (3.2–3.7)	3 (2.7–3.5)^b^	**0.016**
C-reactive protein(mg/dl)	9.16 (3.9–17.5)	5.92 (3.1–14.0)	15.74 (9.6–21.7)^b^	**0.015**
Outcome				
Ventilator-days	16 (8–23)	10 (6.5–16.5)	21 (19–28)^b^	**0.040**
ICU length of stay, days	17 (12–23)	13 (9.5–18.5)	23 (19–30.5)^b^	**0.037**
Hospital length of stay, days	32 (26–51)	29 (25–48.5)	43 (29–66.3)	0.471
Mortality	8 (22%)	1 (4%)	7 (50%)^b^	**0.002**

^a^Categorical variables were presented as frequencies (percentages) and continuous variables are presented as median (interquartile range, IQR). Differences between two groups were analyzed using the Mann–Whitney U test for continuous variables and Fisher’s exact test for categorical variables. Bold values denote statistical significance at the* p* < 0.05 level using Mann–Whitney U test or Chi-square test. ^b^*p* < 0.05 compared with the responders

ICU, intensive care unit; APACHE II, acute physiology and chronic health evaluation II; SOFA, sequential organ failure assessment; COPD, chronic obstructive pulmonary disease

### Distinct dynamic transcriptome in elderly ICU patients with sepsis

**Fig. 1:**
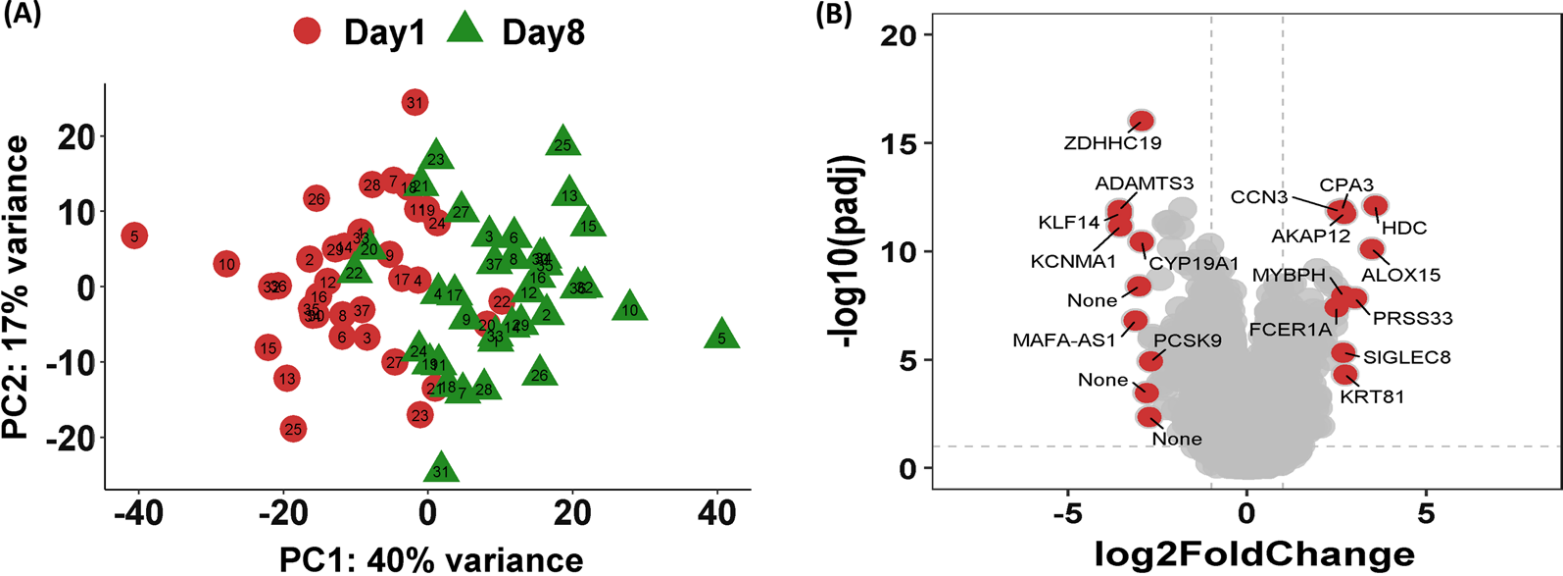
Principal component analysis **A** and volcano plot **B** of the transcriptome between day-1 and day-8

As illustrated in Fig. 1A, using principal component analysis on the top 500 variable genes from all of the samples, we discovered that elderly sepsis patients in the intensive care unit had significantly different transcriptomes on day-1 and day-8. The differential expression genes (DEGs) were then identified by comparing gene expression profiles between day-8 and day-1 in elderly ICU patients using the criteria of *p* < 0.01 and log fold change > 0.25 or < − 0.25. Three DEGs (two upregulated and one downregulated genes) were found in the R group, while the NR group only had 11 DEGs (five upregulated and six downregulated genes). We found that in elderly sepsis patients in the ICU, the top upregulated genes were innate immunity- and inflammation-relevant genes, namely, *ZDHCC19*, *ALOX15, FCER1A*, *HDC, PRSS33,* and *PCSK9*, which bind to low-density lipoprotein receptors (LDLRs), leading to LDLR degradation and increasing LDL cholesterol levels (Fig. 1B).

### DAVID enrichment analysis of the dynamic transcriptome

**Fig. 2:**
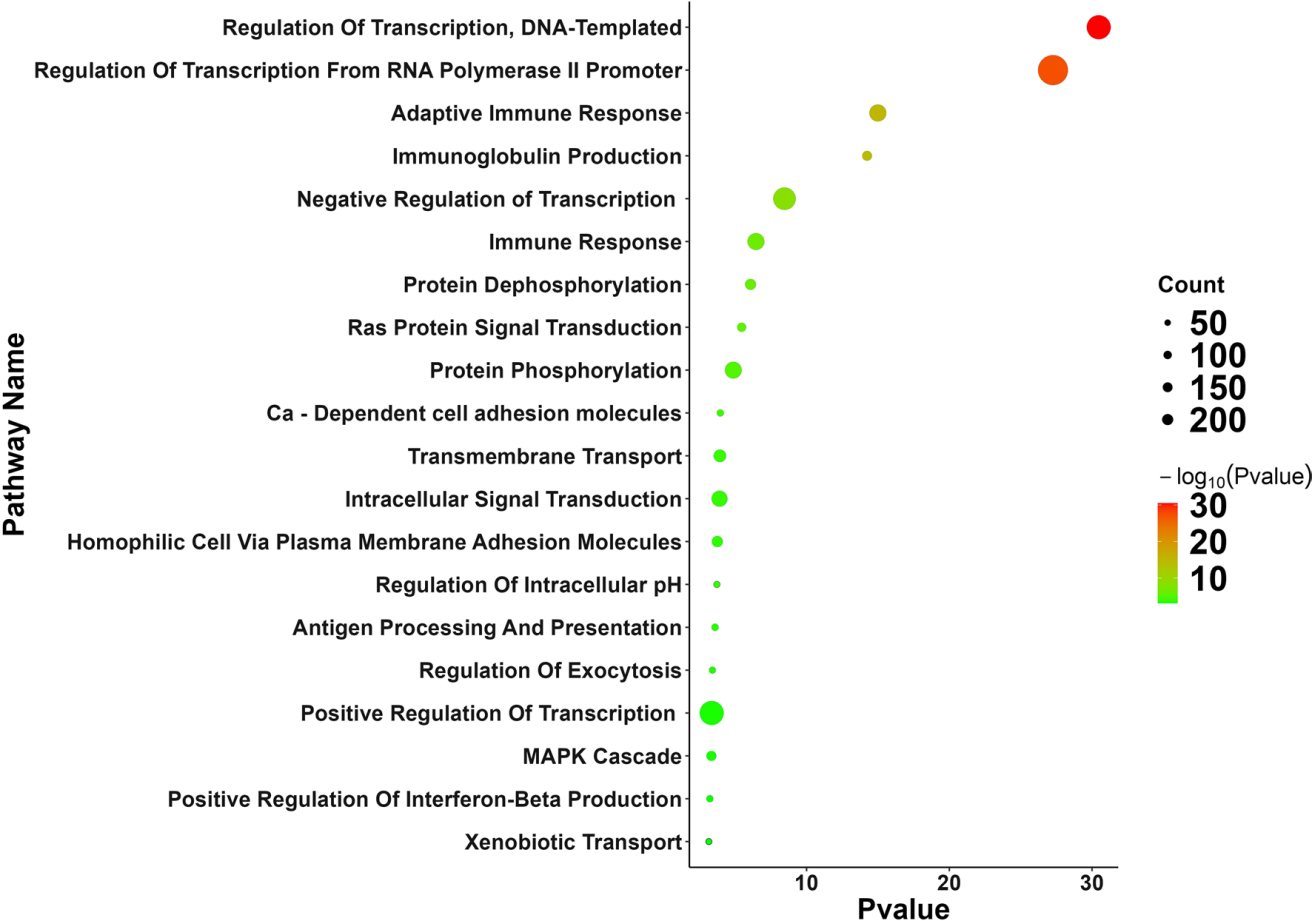
Functional and pathway enrichment analyses of differentially expressed genes with DAVID by bubble plot

**Fig. 3:**
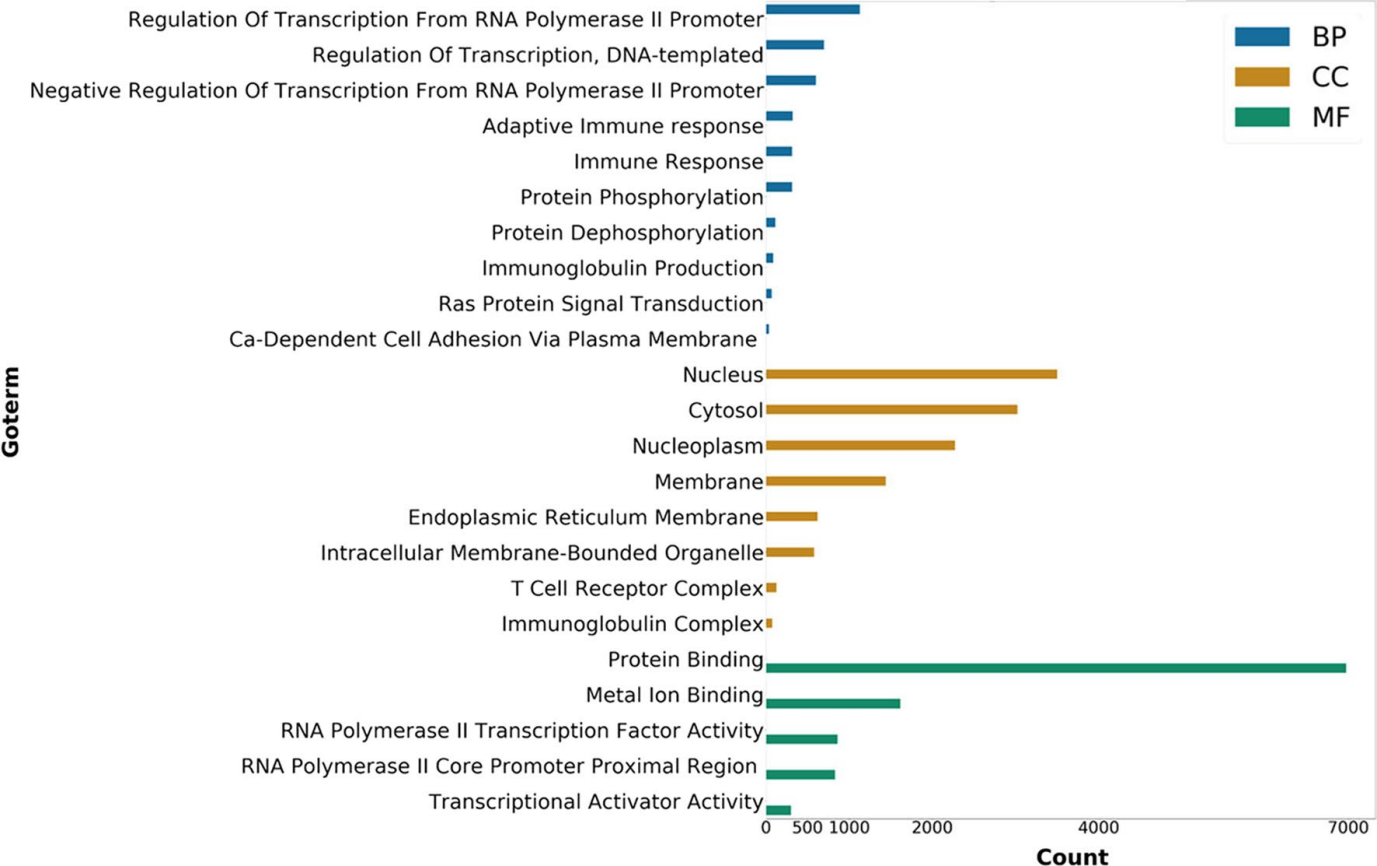
DAVID functional Gene Ontology (GO) analysis of biological process (BP), cellular component (CC), and molecular function (MF)

To demonstrate the alteration of biological pathways in elderly ICU patients between day-8 and day-1, we demonstrated the enriched pathway by utilizing the online DAVID v6.8 server. As shown in Fig. 2, Gene Ontology (GO) bubble plots were constructed for the biological processes of the top 20 DEGs from the dataset. We found that the DEGs from the elderly ICU patients with sepsis were enriched in the regulation of transcription, adaptive immune response, immunoglobulin production, negative regulation of transcription and immune response. DAVID functional GO analysis is presented in Fig. 3. When compared to controls, functional annotations of proteins encoded by genes in DEGs showed significantly (P < 0.05) increased or decreased enrichment. These annotations were categorized according to biological processes, cellular components, and molecular functions. There were significant differences between DEG-encoded proteins involved in the regulation of transcription, the immune response, protein phosphorylation, and Ras protein signal transduction at the level of biological processes (Fig. 3), whereas cellular component analysis revealed significant differences between the T-cell receptor complex, cytosol nucleoplasm, and nucleus (Fig. 3). Significant differences were found in the enrichment of encoded proteins associated with protein binding, meta ion binding, translation factor activity, or transcriptional activator activity according to molecular function analysis (Fig. 3). Then, we used the Cytoscape ClueGO plugin to investigate the functional enrichment of the DEGs in the dataset to further refine the biological process from the analysis of DAVID-GO terms [27]. Additionally, the REACTOME pathway analysis from ClueGO revealed that cytokine signaling in the immune system, adaptive immune system, sensory perception, innate immune system, and immunoregulatory interactions between lymphoid and non-lymphoid cells were significantly enriched in many DEGs.

### TCR analyses with MiXCR in elderly ICU patients with sepsis

Considering that the R and NR groups recovered from sepsis through a specific T-cell receptor signaling pathway, utilizing the number of unique CDR3, D50, and the inverse Simpson index, we used MiXCR to determine the diversity of the T-cell receptor (TCR) (Fig. 5A–C). On day-8, the R group's CDR3, D50, and inverse Simpson index were significantly higher than on day-1. Moreover, the D50 index was significantly higher in the R group than in the NR group on day-8 (Fig. 5B). Finally, we assessed the diversity of unique CDR3 using the inverse Simpson index, which reflects abundant clonotypes. We observed a similar pattern, with significantly increased diversity of TCR in the R group, particularly on day-8 (Fig. 5C).

## Discussion

This study demonstrates that mortality-relevant biological features and adaptive immunity-associated signaling using transcriptomics, comparing differences between day-1 and day-8 (Additional file 1: Fig. S1), could reliably identify elderly ICU patients with sepsis and differentiate unresponsive from responsive patients. Previous studies investigating sepsis biomarkers depended on the selection of known biological functions and pathways using knowledge-based methods [28], and only a small number of studies have directly targeted genes whose functions have not been fully described [29]. In this study, we adopted RNA-Seq to identify biological and immunological features in elderly ICU patients with sepsis through principal component analysis, differential gene expression analysis, and MiXCR. Innate immunity and inflammation, such as *ZDHCC19*, *ALOX15, FCER1A*, *HDC, PRSS33,* and *PCSK9*, were upregulated in elderly ICU patients with sepsis.

ZDHHC19 is a palmitoyltransferase and mediates palmitoylation of RRAS, leading to increased cell viability [30]. Genome-wide gene expression analysis has been linked to increased expression in ICU patients with sepsis caused by fecal peritonitis [31]. HDC (histidine decarboxylase, HDC) is a member of the group II decarboxylase family and forms a homodimer that uses pyridoxal phosphate to convert L-histidine into histamine. An animal study conducted by Hattori et al*.* [32] used *HDC* gene knockout (HDC^−/−^) mice. After inducing sepsis by cecal ligation and puncture (CLP), HDC-knockout mice exhibited reduced plasma histamine levels, and the increase in TNFα, IL‐1b, IL‐6 and MCP1 levels in sepsis were attenuated when there was a lack of plasma histamine [32]. Sepsis-induced abnormal cytokine production and multiple organ injury (lung, liver, and kidney) were significantly reduced in HDC-knockout mice compared to WT C57BL/6 J mice. Inhibition of HDC expression prevented inflammatory tissue injury and improved survival in mice with sepsis induced by CLP [32]. In addition, histamine could promote the production of proinflammatory cytokines, which are regulated by the transcription factor NF‐κB. Under CLP-induced sepsis, HDC-knockout mice had significantly decreased activity of NF‐κB in the nucleus compared with WT mice [32]. This result indicats that histamine induces the synthesis of proinflammatory cytokines and chemokines by increasing NF-kB activity.

Recent research has demonstrated that *ALOX15* and *FCER1A* are crucial to the pathogenesis of bacterial sepsis [33, 34]. *ALOX15*, a lipid peroxidizing enzyme, is a functional lipoxygenase (LOX) gene [35], that oxidizes polyunsaturated fatty acids and has been linked to a variety of physiological processes as well as the pathogenesis of neurodegenerative, inflammatory, and hyperproliferative diseases [35, 36]. ALOX15 and its metabolites are implicated in the pathophysiology of multiple inflammatory diseases, such as sepsis, arthritis, asthma, cystic fibrosis, and atherosclerosis [36]. FCER1A (Fc fragment of IgE receptor Ia, FCER1A), an immune-related protein, is the initiating factor of allergic reactions and plays a role in allergic inflammation [37, 38]. FCER1A has been implicated in regulating metabolic processes and immune regulation in previous studies. [38, 39]. Additionally, asthma is more likely to occur when FCER1B and other immunoglobulin-related inflammatory genes interact [39]. Furthermore, PCSK9 (proprotein convertase subtilisin/kexin type-9, PCSK9) has been identified as a central regulator of plasma LDL-C levels by its ability to bind to LDL receptor (LDLR) and trigger it for lysosomal degradation in cells, leading to LDLR degradation and increasing LDL cholesterol levels [40].

**Fig. 4:**
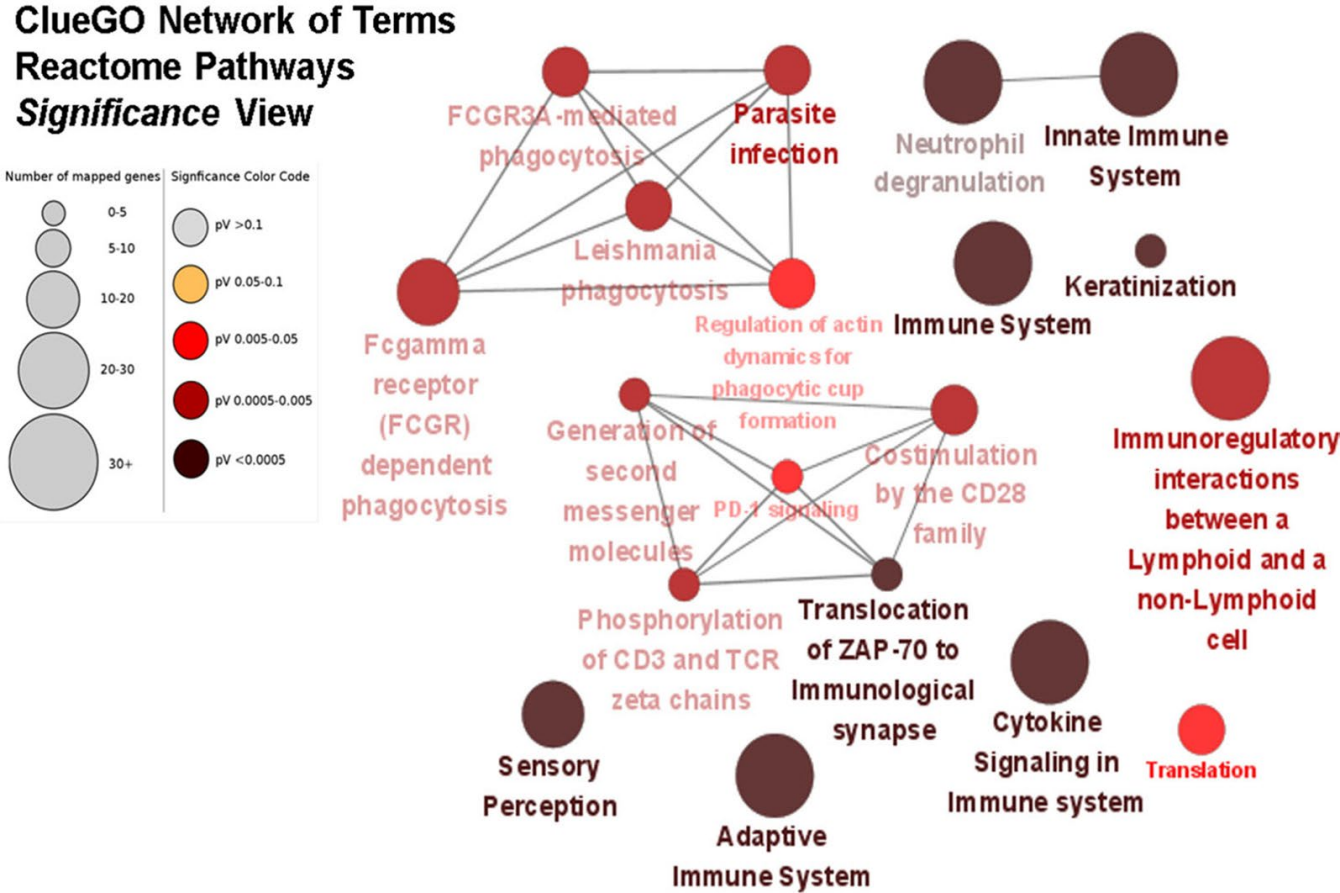
Enrichment by Gene Ontology (GO) terms was visualized using the ClueGO/CluePedia plugin from Cytoscape

In the present study, we used KEGG enrichment and GO to determine the functional annotation of these genes in order to investigate the involvement of expressed DEGs from elderly ICU patients with sepsis in BP, MF, and molecular pathways of FH. These DEGs were primarily enriched in the regulation of transcription, adaptive immune response, immunoglobulin production, negative regulation of transcription and immune response (Fig. 2). We constructed a functionally arranged network of GO/pathway terms using the Cytoscape plugin ClueGO/CluePedia, an improved interpretation for biological terms. This plugin also helped us visualize the networks that are functionally grouped from larger gene clusters [41]. We utilized the ClueGO plugin to identify the differentially regulated molecular pathways and their significant gene interactions based on *p*-values and kappa statistics to obtain a comprehensive picture of the DEGs involved in sepsis. Moreover, the DEGs were significantly enriched in transcription activity and protein binding when MF from GO was analyzed (Fig. 3). These results indicate that functional enrichment analyses refer in particular to immunity cell signaling pathways related to a cellular response to pathogenic motifs or cytokines. These are likely expected results for septic patients and need further investigation to elucidate the pathways in sepsis. Based on kappa statistics and *p-values*, we found that among the enriched biological processes and molecular pathways, cytokine signaling in the immune system, the adaptive immune system, sensory perception, the innate immune system, and immunoregulatory interactions between lymphoid and non-lymphoid cells were dysregulated and were significant associated with the progression of sepsis in elderly ICU patients with sepsis (Fig. 4).

**Fig. 5:**
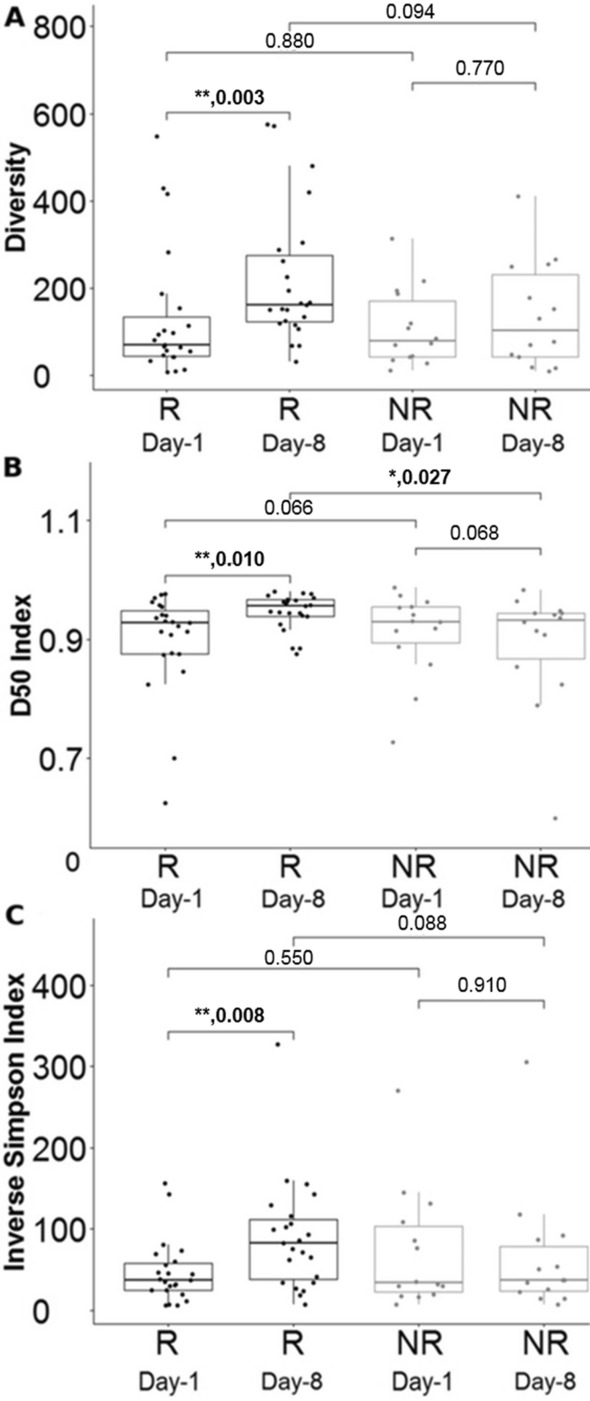
Diversity of the T-cell receptor on day-1 and day-8. Counts (A), unique CDR3 (B), D50 and (C) of inverse Simpson index. * < 0.05, ** < 0.005

Additionally, we showed the diversity of T cells in elderly patients with sepsis and utilized MiXCR to analyze the raw sequence of RNA-Seq to determine clonotypes of TCRs, which made it possible for us to simultaneously examine the diversity of TCRs and the entire transcriptome [15]. RNA-Seq and MiXCR were used in this study to identify T cell signaling and the results revealed that the TCR diversity of the R group on day-8 was greater than that on day-1 (Fig. 5). Compared with the NR group, the R group had significantly higher CDR3, D50, and inverse Simpson index values on day-8, than on day-1. The significance of the induced diversity of TCRs in elderly ICU patients with sepsis is also highlighted by this evidence, which demonstrates the viability of utilizing RNA-Seq and MiXCR data to address the diversity of TCRs in patients with sepsis.

Our study has some limitations that should be noted. First, the group of patients investigated in this study was small. Hence, investigations of diverse populations with a larger sample size would assist in confirming our data. Second, we used the bulk RNA-Seq in this study, and future sc-RNA-Seq and functional experiments are warranted for validation at the cellular level. Moreover, the 5’ rapid amplification of cDNA ends (5’-RACE) approach can be used to investigate the T-cell repertoire, which may further confirm our findings. Third, we assessed the biological and molecular functions of inflammatory responses in patients with sepsis using RNA-Seq data. Further functional studies are required to clarify the roles in sepsis.

## Conclusions

The incidence and mortality of sepsis are both high in elderly individuals. Our study enrolled critically ill elderly individuals with sepsis and identified mortality-relevant biological features and transcriptomic features with functional pathway and MiXCR analyses based on RNA-Seq data. Compared with day-1, we found upregulated innate immunity and increased T cell diversity among responsive patients on day-8, indicating that the responsive patients had better regulated recovery from sepsis compared with the nonresponsive patients. To implement accurate and early prediction, utilizing a test of RNA-Seq could offer additional complementary information for the early prediction of treatment outcome.

## Supplementary Information

**Additional file 1: Figure S1.** Principal component analysis of day-1 (A) and day-8 (A) transcriptome in the response patients (responder group, R) and without response patients (non-responder group, NR) with septic.

## Data Availability

The original contributions presented in the study are included in the article and further inquiries can be directed to the corresponding author. The original contributions presented in the study are publicly available. This data can be found here: https://www.ncbi.nlm.nih.gov/geo/query/acc.cgi?acc=GSE216902.

## Abbreviations

RNA-Seq: RNA sequencing
ICU: Intensive care unit
DEGs: Differentially expressed genes
TCRs: T-cell receptors
SOFA: Sequential organ failure assessment
CDR3: Complementarity-determining region-3
GO: Gene ontology
CLP: Cecal ligation and puncture
