# Supplementary material for: Whole transcriptome analysis to explore the impaired immunological features in critically ill elderly patients with sepsis
Source: J Transl Med. 2023 Feb 23;21:141. doi: 10.1186/s12967-023-04002-z (PMC9951485; doi:10.1186/s12967-023-04002-z)
Supplement: Supplementary file 1 — Additional file 1: Figure S1. Principal component analysis of day-1 (A) and day-8 (A) transcriptome in the response patients (responder group, R) and without response patients (non-responder group, NR) with septic. [file 12967_2023_4002_MOESM1_ESM.docx]

Additional Material

# Additional Figures

**
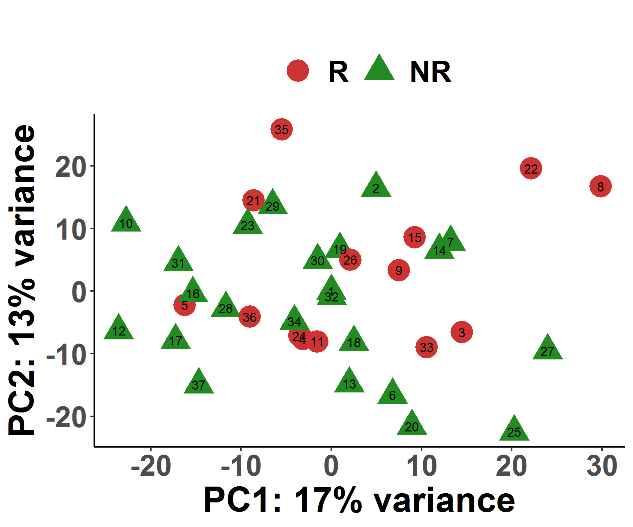

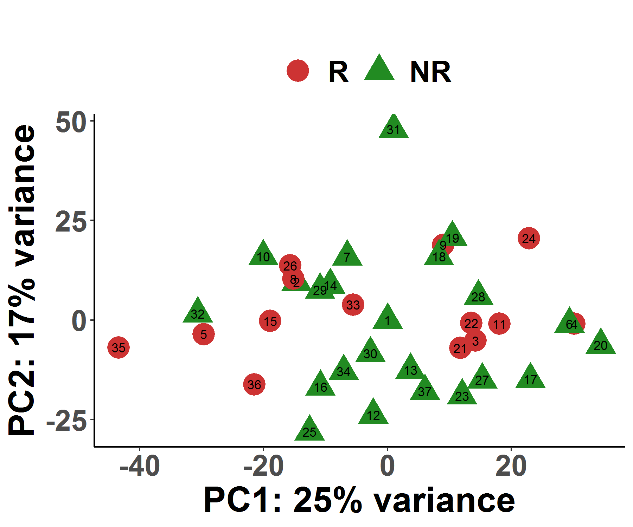
**(A) Day-1 (B) Day-8

**Supplementary Figure 1.** Principal component analysis of day-1 (A) and day-8 (A) transcriptome in the response patients (responder group, R) and without response patients (non-responder group, NR) with septic.
